# Extended editorial: preventing fraud and cybercrime in an ageing society

**DOI:** 10.1057/s41284-025-00479-z

**Published:** 2025-06-21

**Authors:** Mark Button, Vasileios Karagiannopoulos, Julak Lee, Joon Bae Suh, Jeyong Jung

**Affiliations:** 1 Centre for Cybercrime and Economic Crime, University of Portsmouth, Portsmouth, UK; 2 Chung Ang University, Seoul, South Korea; 3 Korean National Police University, Asan, South Korea; 4 University of Ulsan, Ulsan, South Korea

## Introduction

**Fig. 1:**
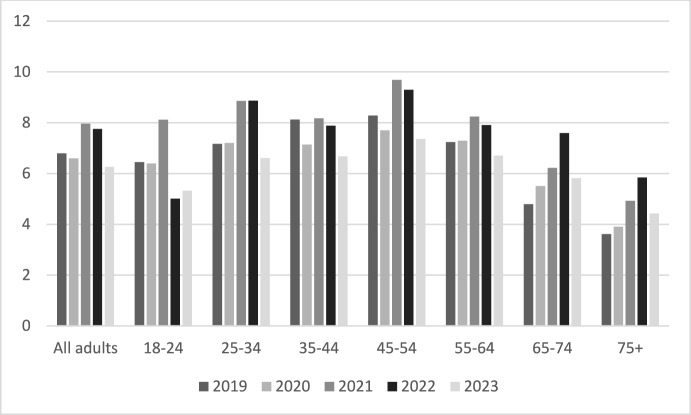
Percentage of age groups victims of fraud in England and Wales. Note, from 2023 the 18–24 age group expanded to 16–24. ONS (2024)

The nature of crime has been changing globally with technological and other societal developments fuelling a growth in fraud and cybercrime (Button and Cross 2017). The diversity and rapid evolution of a broad range of scams has led to millions across the globe becoming victims of fraud (Federal Trade Commission 2019; ONS 2023a, 2023b). A common belief often promoted in policy circles and some research is that older adults are more vulnerable to fraud (James et al. 2014). This is not so clear cut, however, as in terms of victimisation, the middle aged have been most at risk (see Fig. 1 later). This is changing and there is evidence older adults (65 +) are fast becoming the one of the most at risk categories and that they also tend to lose much more than other age groups (see Table 1 later). Indeed, there are a variety of trends fuelling a potential explosion in fraud and cybercrime among older adults unless serious action is taken to reduce the risks that we will shortly explore. It is for this reason we conceived this special edition and the research project that underpins it.

**Table 1:** Average losses by age group reported to Action Fraud 2020–2022

Group 2020 2021 2022			
Age group	2020	2021	2022
18–24	£1251	£2922	£2052
25–34	£2189	£2554	£2970
35–44	£3724	£3995	£5135
45–54	£5868	£5605	£7135
55–64	£9229	£8445	£9392
65–74	£12,715	£9972	£10,231
75 +	£13,942	£13,428	£13,112

FOI request to action fraud

To set the context for this edition, it is important to examine the factors fuelling the growth in fraud and cybercrime victimisation among older age groups. We will do this using data from the UK and South Korea. The reason for the selection of these countries is simple. The funding for the project stimulating this edition came from ESRC funding aimed at developing relationships with these two countries on issues such as ageing and technology. However, they are both highly developed countries with ageing populations experiencing a rise in fraud and cybercrime, so although the main reason is purely funding, they do provide two very useful case studies to illustrate the challenges and problems of ageing, fraud and cybercrime. The special edition, however, has papers from a much wider range of countries, including the USA, Australia, Brazil, UK, India and Nigeria too.

### Older adults: fraud and cybercrime

Frauds and scams are often used interchangeably to describe deceptive behaviours that cause a person some form of financial loss. For instance, Button and Cross (2017) note scams can be deceptive behaviours which cause a loss, which are clearly unethical, but lawful; whereas frauds are always unlawful. Another term important to grasp in this context is financial abuse, which is also a term with much debate on the scope, but generally considered to cover the improper or illegal exploitation or use of funds/resources of an older person (Fealy et al. 2012). It is also usually more associated with those close to the older adult such as family, carers or professionals working with them. Cybercrime is a much broader concept that covers both economic, psycho-social and state cybercrimes (Ibrahim 2016). This editorial and edition is only interested in economic cybercrimes: cyberfrauds, ransomware and hacking for monetary purposes, with a particular focus on: frauds, scams, financial abuse and economic cybercrime.

There are a number of methods of fraud and cybercrime which are common for older adults and types of fraud. Some of the more common include doorstep frauds/scams: rogue tradesmen who massively overcharge for their services and/or conduct works not required or to a poor standard. Door-to-door sales of non-existent or low quality services and/or goods (Phillips 2017). Financial abuse of older adults by carers, relatives or friends (Dalley et al. 2017). Telephone scams, where there is high pressure sales of worthless goods or service, particularly investments; impersonation scams that trick victims into transferring money and vishing, where sensitive personal information is sought (Choi et al. 2017; Lee 2020). Postal scams which utilise fake lotteries, bogus charities and investment schemes to name some touted through traditional mail (Rebovich & Corbo 2021). The Authorised Push Payment (APP) frauds where the victim is deceived into authorising a payment to a criminal via impersonation (National Fraud Authority 2011; Age UK 2023). Identity frauds: where victims are impersonated to use their financial credentials or identity fraudulently (Deliema et al. 2021; Cifas 2022). Finally, cyberfrauds: a wide range of scams using email, the internet and social media (Age UK 2017; Button and Cross 2017). The types of frauds and scams which particularly target older adults include:

- Consumer related frauds: sales of goods and services which are non-existent or worthless (Jorna 2016; Fan and Yu 2021).
- Investment related frauds: sales of bogus or overpriced investments (Deliema et al. 2020a, b).
- Romance and relationship frauds: bogus lovers and friends online who build up trust with aim to secure money from victim (Deliema 2018; Bailey et al. 2021).
- Impersonation of police/security frauds: fraudsters impersonate police and/or bank security staff with aim to trick victim into giving money or sensitive financial credentials away (Payne 2020; Benbow et al. 2022).
- Phishing-related frauds: a wide range of frauds seeking personal information related to victim (Choi et al. 2017; Button et al. 2021; Benbow et al. 2022).

In the previous years, COVID-19 also generated an additional layer of scams and financial abuse with safeguarding agencies reporting that community-dwelling adults had been targeted by pandemic-related scams regarding COVID-19 safety-related products. Risks for older adults have thus been exacerbated by the unavoidable increase in the use of technological tools for shopping, healthcare etc., which has exposed this population to increased risks from cybercriminals, whilst making traditional risk assessments and safeguarding efforts more complex (Hakak et al. 2020; Benbow et al. 2022). Many older adults are lucrative potential targets, as large numbers have lifesavings, retirement ‘pots’ and investments.

In the past few years, COVID-19 has also exacerbated ageist discourses further enhancing perceptions of increased vulnerability of older adults and thus potentially highlighting them as ideal targets for criminals, particularly due to the increased isolation afforded by the pandemic, which distanced them even further from family and support networks (Han and Mosqueda 2020).

It is also clear from the literature that the impact of fraud on older adults can be devastating with not only the financial loss, but also psychological, physical and mental health and even suicide to name some (Button et al. 2014; Cross 2016b; Deliema et al. 2020a; Bailey et al. 2021; Button et al. 2021, 2024a, b). Research from Korea has also found that as the number of people aged 65 or older and the elderly living alone increases, frauds also tend to increase, and frauds also affect the suicide rate in the following year. The researchers collected crime and administrative data from all 215 regions in South Korea for five years from 2015 to 2019, built panel data and analysed them using fixed effect models. According to the study, frauds tend to increase as the population ages, and those over 65 or those living alone are more likely to be victims of fraud than other age groups. It seems that the ability to respond to scammers who use highly intelligent methods declines as the elderly population increases. Since the relationship between fraud victimization and suicide is highly likely to occur in the older adults, the researchers argued it is necessary to introduce fraud prevention education (Cho et al. 2021). Reports in the news in the UK in February 2023 also suggested fraud in England and Wales would be upgraded to a national security threat, such is the impact of it, with evidence that the crime puts 300 people a year in such a state of harm they are a suicide risk (Telegraph 2023).

### Fraud trends

In recent years, older adults in England and Wales have become more likely to become victims of fraud. Figure 1 below illustrates the fraud victimisation rates across a range of age categories from 2019 to 2023. Victimisation rates increased across all age categories during the pandemic but, excluding that period, have remained relatively stable with the exception of the 65–74 and 75 + age groups, where there has been a continuing upward trend.

Older adults may not be the most at risk group, but they tend to lose more. Table 1 below shows as the age rises so does the average losses, with the 65–74 and 75 + losing the most. Many of these are in positions in life where it is harder to recover from losses, dues to fixed pensions.

The risk of being a victim of fraud is also increasing for older adults in South Korea. In 2018, there were about 267,000 fraud crimes nationally, which peaked at about 345,000 in 2020, and the most recent figure is about 325,000 in 2022. The overall number of fraud crimes has shown an upward trend, even though the number of fraud crimes between 2019 and 2021 has fluctuated. On the other hand, the number of victims of fraud among those aged 60 and over is increasing more rapidly. In 2018, the number of cases of older adult victims increased from about 20,000 to about 46,000 in 2022, showing a steady upward trend. If we calculate the proportion of fraud crimes against older adults in the total number of fraud crimes, it is found that the proportion has more than doubled in four years, increasing from about 7.5% in 2018 to about 14.2% in 2022. This means that whilst the number of frauds against older adults is increasing not only in absolute numbers, it is also increasing as a percentage of all frauds.

### Ageing in the UK and South Korea

Ageing in itself does not cause greater fraud, but there are additional factors associated with it that do. Before these are explored it is important to consider the significant ageing of the UK and Korean populations which is taking place. In the UK by mid-2020, there were 1.7 million people 85 + years—2.5% of the UK population. By mid-2045, this is projected to nearly double to 3.1 million, 4.3% of the total UK population (ONS 2022b). In South Korea, the proportion of the population aged 65 or older was 15.7% in 2020, will be 20.3% in 2025, and 25% by 2030 and it is expected that the current ageing society will enter a super-aged society (South Korea. Korean Statistical Information Service. 2023). Table 2 below illustrates the large numbers of older adults in the UK and South Korea across older adults age categories with in the UK around 15 million and in South Korea almost 12 million.

**Table 2:** Total number of older adults in the UK and South Korea

Age group	UK	South Korea
60–64	3,855,818	4,067,484
65–69	3,355,381	3,066,136
70–74	3,363,906	2,145,118
75–79	2,403,759	1,551,840
80–84	1,726,223	1,201,647
85–89	1,049,866	777,432
90 and older	609,503	
Total	16,364,456	12,809,657

ONS (2022a, b, c) and KOSIS (2023)

For UK data is 2021, for South Korea 2022

### Macro factors fuelling fraud

#### Health of older adults

An ageing population brings with it some significant health challenges with larger numbers of older adults experiencing health conditions which may increase their risk of becoming fraud victims. These include dementia, declining mental and physical health to name some. Declining cognitive ability has been linked in several studies to increased risk of fraud (James et al. 2014; Duke Han et al. 2015; Judges et al. 2017; Ueno et al. 2021). Severe dementia is not the largest risk of fraud because by this point the individual usually has a third party looking after their financial affairs (although this does open up a bigger risk of financial abuse). Mild and moderate dementia are seen as the greater risk to fraud victimisation as at this stage they are likely to control their financial affairs and their cognitive decline may make them less resilient to frauds and scams (Ueno et al. 2021). In the UK in 2019, the number of older adults with mild dementia was 126,900 and with moderate, 245,600. By 2040, this is set to increase by 55% for mild to 196,000 and by 33% for moderate to 327,500 (Wittenberg et al. 2019). In South Korea, the estimated number of people aged 65 and over with dementia as of 2020 was 840,000, with an estimated dementia prevalence rate of 10.3% (National Institute of Dementia 2023). This means that 10 out of every 100 people aged 65 and over are estimated to have dementia. The number of patients with mild cognitive impairment, a pre-dementia stage, also exceeded 150,000 and 357,506 with moderate dementia in 2020 (Ministry of Health and Welfare 2022). According to the National Institute of Dementia (2023), the estimated number of dementia patients in Korea is expected to reach 1.36 million in 2030 and exceed 3 million in 2050.

Burton et al. (2022) highlight one fifth of people aged 65 + experience some level of cognitive impairment, with many of those being Internet users opening up opportunities for a wide range of fraud victimisation. Mental illness is also a problem among older adults. In the UK, depression affects around 22% of men and 28% of women aged 65 years and over. It is estimated that 85% of older people with depression receive no help at all from the NHS. It is estimated 7–17% of older adults suffer from social isolation and this is set to continue to grow (Health and Social Care Information Centre 2007). As was noted earlier depression has been linked to greater risk of fraud victimisation (Lichtenberg et al. 2013; Burnes et al. 2017; DeLeima et al. 2020a, b).

Physical health is also an important factor because a decline can decrease mobility, increase social isolation and mean greater time spent at home. All of these factors are associated with increased risk of fraud (Ross et al. 2014; Burnes et al. 2017; Burton et al. 2022). High blood pressure among men has also been linked as a factor linked with increased fraud victimisation (Lamar et al. 2022).

In the UK, an estimated 4 million older adults in the UK (36% of people aged 65–74, and 47% of those aged 75 +) have a limiting long-standing illness, equating to 40% of all people aged 65 + (Horsfield 2017). Those living with frailty are at greater risk of disability, care home admission, hospitalisation, and death (Wirral 2018). Approximately, 3% of the population aged 65 + in England live with severe frailty, 12% with moderate frailty and 35% with mild frailty (BMA 2018). As age rises, the proportion of people living with frailty increases: 6.5% in those 60–69; 65% in those 90 + (Gale et al. 2015). Mobility difficulties are also common among those living with frailty (93% have difficulties vs only 58% of non-frail individuals) (Gale et al. 2015). The ageing trends means this will increase rates of frailty further.

In South Korea, the situation is similar. Among older adults 37.0% thought, they were healthy, and 39.7% thought they were unhealthy (Lee 2018). More than half of older adults live with 3 or more chronic diseases, and the rate of 2 or more is 73.0%. About 3/4 of the elderly have overlapping chronic diseases (Lee 2018). Approximately, 89% of older adults in South Korea suffer from one or more chronic disease and considering Korea’s life expectancy of 82.7 years and healthy life span of 64.4 years, older adults are predicted to live in an unhealthy state for about 10 years or more (South Korean Ministry of Health and Welfare 2017). In 2017, more than 40% of patients who received hospital treatment for depression were aged 60 or older. Among them, 30.2% of the elderly living alone, 16.4% of the elderly living with a spouse and 21.7% of the elderly living with adult children experienced depression (South Korean Ministry of Health and Welfare 2017; Won and Kim 2020).

#### Living alone and older adult households

Loneliness and/or living alone has also been linked with increased risk of fraud victimisation (Alves and Wilson 2008; Peterson et al. 2014; Cross 2016b; Xiang et al. 2020; Wen et al. 2022), along with the associated issues of lack of social networks, family, friends etc. (Deliema 2018; DeLeima et al. 2020a, b). England and Wales has a large number of households with those 66 and over living alone at 3.2 million (ONS 2022b). In South Korea, there is also a trend towards greater numbers of older adults living alone. The proportion of older adults living alone has increased from 16.0% in 2000 to 20.8% in 2022 amounting to 1,875,270 older adults (Statistics Korea 2022). Living alone and social isolation have also been linked with increased risk of fraud victimisation (Cross 2016a; Deliema 2018; DeLeima et al. 2020a, b).

#### Use of technology

There is much research linking routine activities theory to fraud victimisation (Holt and Bossler 2008; Reyns 2015; Leukfeldt & Yar 2016). Naturally the more time a person spends doing something the greater chance they will stumble across a situation that provides an opportunity for a fraudster to exploit. Thus more time spent shopping online increases the risk of shopping fraud, dating online leads to romance fraud, banking online lead to online banking fraud to name some. There are significant numbers of older adults who do not engage in these activities at present, but this looks set to change with increased opportunities for fraudsters to exploit with older adults with limited digital skills.

The use of the internet is a key vector towards fraud and cybercrime. Over the last decade there has been a substantial increase in the use of the internet among older adults in the UK. In 2013, 3.5 million of 65–74year olds had used the internet in the past three months, had grown to 5.5 million in 2020 and for 75 + the increase had gone from 1.3 million to 2.9 million during the same period (ONS 2021). Perhaps even more significant is that for the numbers who had never used the internet; for 64–74 year olds fell from 1.9 million to 736,000 and for 75 + 3 million to 2.1 million between 2013 and 2020. In South Korea for those aged in their 60 s, the internet usage rate increased by 12.0% to reach 94.5% and in the 70 s and older age group, it increased by 17.9%, approaching half (49.7%) of all households (National Information Society Agency, 2022).

The use of smartphones and related technologies has also increased among older adults. Just between 2018 and 2019, the percentage of older adults aged 65–74 increased from 40 to 43% and for 75 + , 14% to 23%. In 2019, 25% of 65–74 year olds possessed a mobile phone with access to the internet and 13% of 75 + . The possession of a tablet rose from 19 to 26% for 75 + , but fell from 42 to 36% for 65–74 year olds. However, for both age groups there was a rise in the ownership of desktop computers, rising from 8 to 13% for 75 + and 19–23% for 65–74 year olds (OFCOM 2020). In South Korea, 83% of 60 + year olds use a smartphone (Gallup 2021). It seems that the use of smartphones among the elderly in South Korea is very high.

There is also evidence in the UK of a lack of skills among many older adults to use the internet and related technologies safely. Research has found limited knowledge of privacy and security threats—issues with digital literacy, with some more isolated and less able to engage in peer learning networks or assess quality advice (Tennant et al. 2015). The high fear of crime and perceived high susceptibility to threats can lead to disengagement with security measures and lower interaction with online services (Roberts et al. 2013; Nicholson et al. 2019). Older regular internet users are also not confident using the internet for finding security information or resolving security/technical situations—language challenges (Nicholson et al. 2019; Karagiannopoulos et al. 2021). There is also ignorance of reporting mechanisms—consequently low levels of reporting (Karagiannopoulos et al. 2021; Nicholson and Glasson 2020).

#### Wealth of older adults

Many older adults are also attractive targets as they have built up wealth during their working lives. In the UK between April 2018 and March 2020, the average wealth of someone 75 + was £271,800, 64–75 was £338,100, falling to £253,600 for the 45–64 age category, then to £52,000 for the 25–44 age category and only £15,100 for those in the 16–24 category (ONS 2022a, b, c). According to Korea’s 2022 wealth statistics by age group, those aged 60 and over had KRW 483 million (£289,121), those aged 50–59 had KRW 534 million (£319,907), those aged 40–49 had KRW 469 million(£280,661), those aged 30–39 had KRW 299 million (£179,107) and those aged under 30 had KRW 84 million (£50,750) (Bank of Korea and Financial Supervisory Service 2022). Those in their 60 s and older owned fewer wealth than those in their 50 s, but more than any other age group.

**Fig. 2:**
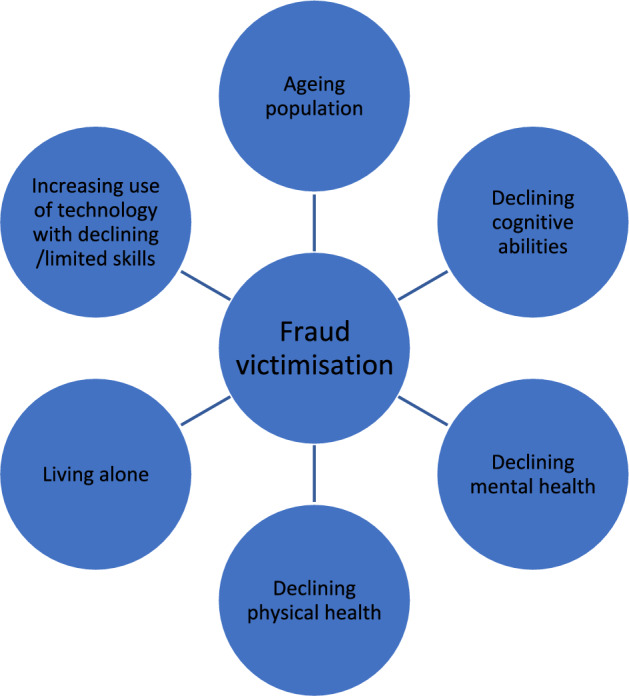
The fraud pressures for older adults

Figure 2 above brings together the discussion above to illustrate the many factors associated which have been linked either directly to fraud or to factors associated with increased likelihood of victimisation. Some may be less important than others, but all are likely to have some impact on fraud victimisation and this illustrates the need for more research to truly appreciate the influences upon fraud victimisation among older adults and more generally. The section has also shown the strong push factors towards greater risks of fraud and cybercrime victimisation among older adults.

### The future of fraud

Predicting the future is a notoriously difficult task, as Niels Bohr argued “Prediction is very difficult, especially if it’s about the future” (Cranfield 2013). There are however technological changes unfolding that when assessed in the context of previous fraud, one can make a reasonable assumption of potential new types of fraud. These will now be explored.

#### Fraud narratives

The most difficult to predict in the medium to longer term are the fraud narratives. Fraud narratives are constantly change and they tend to mirror more broader events and trends in wider society. So the energy price hikes following the Russian invasion of Ukraine triggered scams related to securing grants/refunds etc. related to energy bills. During the pandemic, there were a variety of scams that merged linked to it such as bogus protective equipment, government support grants etc. (Kemp et al. 2021). The narratives of frauds are constantly changing and it is difficult to predict what these will be in the medium to longer term, but one can be attuned as a significant problem or event unfolds that fraudsters will be looking to exploit it. It is therefore futile trying to predict future fraud narratives above the immediate short-term. There are, however, other factors which we can be more confident of predicting as they relate to advances in technology which can be used to perpetrate frauds with the appropriate fraud narratives of the future.

#### AI-related fraud

AI has been written about extensively in relation to the potential to detect frauds. It has already and is likely to be increasingly used to perpetrate frauds against older adults and many other sections of the community.

(i) AI as selection tool

AI is likely to be used to select the most vulnerable victims. Scammers may use it to refine their selection criteria so they do not have to target as many victims and can be more confident that those they target will be more likely to become victims.

(ii) AI as content creator

AI through large language models like ChapGPT is already being used and likely to be used much more by fraudsters to write more persuasive scripts that are most likely to be successful with different types of victims (Europol 2023). Fraudsters with poor English will be able to develop much better quality text tailored to the victims and perhaps with more convincing arguments and related language. So, the old advice to look for poor grammar and spelling mistakes will be useless, whilst at the same time, the messaging will entail a higher degree of manipulative/social engineering potential. But more significantly fraudsters will be able to ask AI to write a persuasive request for a particular audience: women over 60, teenagers etc.

(iii) Realtime advisor

Linked to the above it will also be possible for fraudsters to develop real-time tools where scammers on the phone or chatting can use it to advice on most appropriate responses from victims. Again they will be able to tailor the responses by setting parameters to answer questions as if from a specific demographic.

(iv) AI an impersonator

AI is already been used in a wide range of scams to impersonate the voices and images of not just famous individuals but the loved ones of targets. This is likely to occur on a much larger scale. Scammers have already impersonated celebrities to orchestrate scams. In the UK, the leading consumer rights campaigner, Martin Lewis, was impersonated in a deep fake video which endorsed investments he did not (Money Saving Expert 2023). In China, scammers used deep fake technology to create videos to impersonate government officials asking potential victims to transfer monies to them. In India, scammers used such technology to change voices to impersonate officials to target victims by telephone asking them to transfer monies (ACFE 2023).

Perhaps of even more concern will be the potential of AI to impersonate friends, family and work colleagues to trick them into giving money or information. Spear phishing targeting specific individuals has become a common scam, but AI will enable this to be taken to the next level as tools become freely available and widely used that enable fraudsters to impersonate the voices and eventually the images of associates of potential victims and then contact them to try and secure sensitive information or payments. Such frauds have already occurred when a Chief Executive’s voice was cloned to trick his company into making a US$220,000 payment (Wall Street Journal 2019). Researchers from Greece recently demonstrated the potential for deep fakes to trick virtual assistants to disclose personal information of their owners or engage in monetary transactions on their behalf, highlighting the need for enhanced security measures for VAs and more awareness on the side of users (Bilika et al. 2023).

The threat of these deep fakes will become more potent as AI is used to develop convincing voice messages for vishing, a threat particularly common for older adults and even video calls, which can make the scam even more convincing. The example of VALL-E voice cloning software is indicative of how deep fake voice messages can be developed with only seconds of a voice sample from someone and can then be used to breach bank accounts or get money from friends and family (Hauser 2023).

#### Metaverse

Another space where online fraud is expected to flourish in the coming years is the Metaverse and generally VR environments. Although the Metaverse transition seems to be slower than what Mark Zuckerberg expected, resulting in major losses for Meta, it seems that Meta environments, as currently heavily unregulated spaces could become fertile ground for old and new forms of fraud. According to Gartner Inc (2022): “By 2026, 25% of people will spend at least one hour a day in the metaverse for work, shopping, education, social and/or entertainment” whilst its economy will be dominated by cryptocurrencies and non-fungible tokens (largely unregulated), whilst many organisations are expected to gradually develop products and services for the Metaverse. In this environment, Smaili and De Rancourt-Raymond (2022) highlight that there can be a variety of considerations for cyberattacks, privacy breaches and identity theft. Mystakidis (2022) underlines the risk of tracing Metaverse avatars back to their offline users and the potential for social engineering through the collection of avatar-related information of victims by more experienced users. According to Kadar(2022), the largely unregulated nature of cryptocurrencies and NFTs, will also generate opportunities for associated scams and fraud, such as phishing for NFT account hacks.

#### Exploiting technological skills deficits

As more and more services move online and it becomes increasingly difficult to access services such as banking, customer service, online accounts etc. in-person or even on the telephone, there will be a minority of persons with limited skills who will struggle to access services, particularly the elderly or individual with disabilities (Karagiannopoulos et al. 2021). For example, a study found that older adults demonstrated anxieties linked with adopting new technologies and feelings of “inferiority and powerlessness” caused by a lack of technological expertise (Wu et al. 2015). The European Commission 2020 Eurobarometer report on Europeans’ attitudes towards cyber security (cybercrime) also highlighted that only 36% of those aged 55 and older feel well informed about the risks of cybercrime with just 14% of the same age group being aware of existing channels for reporting cybercrime. Consequently, we can expect that as we are transitioning to environments that require more in-depth awareness of technology and involve more advanced and perhaps even yet unknown victimisation risks, individuals lacking the capability or familiarisation with new technologies will be further disadvantaged at preventing and dealing with technology dependent or facilitated scams and other cyberattacks.

## Introducing this edition

The discussion above has made clear the risk to older adults of fraud and cybercrime in the future from both a growing proportion of the population exhibiting traits that put them at more risk of victimisation, juxtaposed to a number of factors that will make the potential frauds that can be developed more potent.

This edition was framed from an ESRC funded research project directed at improving relations between the UK and South Korea on a research topic related to ageing and technology. The investigators, who are also the authors of this editorial embarked on a variety of research projects, some of which have already been published (see Button et al. 2024a, b, 2025a). As part of the project seminars and conferences were held, there was also an objective to develop a special edition of a journal dedicated to this subject. This edition is the result of that containing 12 papers from writers not just from the UK and Korea, but the USA, Australia, Brazil, China, Nigeria and India too.

The edition includes an impressive range of papers exploring the modus operandi and the factors of victimisation regarding fraud and cybercrime against older adults in countries where there is limited research on this issue such as India, Nigeria, Brazil, China and South Korea (Datti et al. 2025; Kim et al. 2025; Lazarus et al. 2025; Porto-Bellini 2025; Shapiro 2025; Uroko and Obiorah 2025), as well countries such as the USA and Australia where there is already much research (Cross and Holt 2025; Viana et al. 2025). The edition highlights the problem of fear of fraud for older adults, widely noted for many other crimes, but not for fraud (Button et al. 2025b). Very importantly the edition includes cutting edge papers illustrating theoretical and practical prevention-focussed analyses using both technology-based solutions (Robinson and Edwards 2024; Qiu 2025), as well as more traditional policies (Lee et al. 2025; Parti 2025).

This editorial has noted fraud and cybercrime against older adults looks set to be a significant problem for the future. This edition’s papers provide a strong foundation for the debates and research on this issue that will no doubt emerge in coming years. It is only a start and much more research is required, but we hope together it stimulates ideas and most of all you enjoy it!
